# Increased NF-L levels in the TDP-43^G298S^ ALS mouse model resemble NF-L levels in ALS patients

**DOI:** 10.1007/s00401-022-02436-1

**Published:** 2022-05-18

**Authors:** Eva Buck, Patrick Oeckl, Veselin Grozdanov, Verena Bopp, Julia K. Kühlwein, Wolfgang P. Ruf, Diana Wiesner, Francesco Roselli, Jochen H. Weishaupt, Albert C. Ludolph, Markus Otto, Karin M. Danzer

**Affiliations:** 1 German Center for Neurodegenerative Diseases (DZNE), Ulm, Germany; 2 Neurology, University Clinic, University of Ulm, Ulm, Germany; 3 Division for Neurodegenerative Diseases, Neurology Department, Mannheim Center for Translational Neuroscience, University Medicine Mannheim, Heidelberg University, Mannheim, Germany; 4 Department of Neurology, Martin-Luther-University Halle-Wittenberg, Ernst-Grube Str. 40, 06120 Halle (Saale), Germany

Elevated levels of neurofilament light chain (NF-L) in CSF and blood are linked to the presymptomatic and symptomatic phase of patients suffering from amyotrophic lateral sclerosis (ALS). However, whether the NF-L level in extracellular liquids like serum or CSF is a marker of destruction or NF-L is secreted actively outside the cell is not known so far. NF-L levels in CSF and blood clearly separate ALS patients and controls [9], serving as a prognostic biomarker for ALS [4]. Also in pre-symptomatic ALS gene mutation carriers NF-L levels are elevated thus allowing prediction for clinical phenoconversion [3, 4]. The picture of NF-L levels in ALS mouse models is less clear. Previous studies report on elevated NF-L plasma levels in SOD1^G93Adl^ and TDP-43 (TAR6/6) mice [6], but the correlation to motor neuron (MN) loss has not been determined. We therefore employed a transgenic TDP-43^G298S^ mouse model to study the interaction of motor neuron pathophysiology, muscle denervation and NF-L levels. TDP-43^G298S^ mutant mice show decreased performance in grip strength and motor activity compared to controls [10].

Serum NF-L level measurements were carried out with the ultrasensitive single molecule array (Simoa) technique and showed a significant age- and genotype-dependent increase of NF-L in both WT and TDP-43^G298S^ mice (****p < 0.0001 for age, **p < 0.01 for genotype, Two-Way ANOVA with Tukey’s multiple comparison test) (Fig. 1a). In contrast to WT mice TDP-43^G298S^ mice had increased NF-L levels already at 2–3 months of age that continuously raised until reaching a plateau at 8 months (***p < 0.001 for 2 months, *p < 0.05 for 8 months, *p < 0.05 for 18 months; Mann–Whitney test comparison WT vs TDP-43^G298S^). WT mice needed 12 months longer to reach the same plateau as TDP-43^G298S^ mice, thus potentially pointing to a premature aging phenotype or to early MN degeneration in TDP-43^G298S^ mice. These findings are in line with previous studies demonstrating increased NF-L levels at 9 and 27 weeks of age in transgenic TDP-43 (TAR6/6) and SOD1^G93A^ mice, respectively [6]. From 10 months onward, NF-L levels are constantly high in our TDP-43^G298S^ mouse model compared to controls and are in line with clinical data from ALS patients [3, 4, 8].

**Fig. 1:**
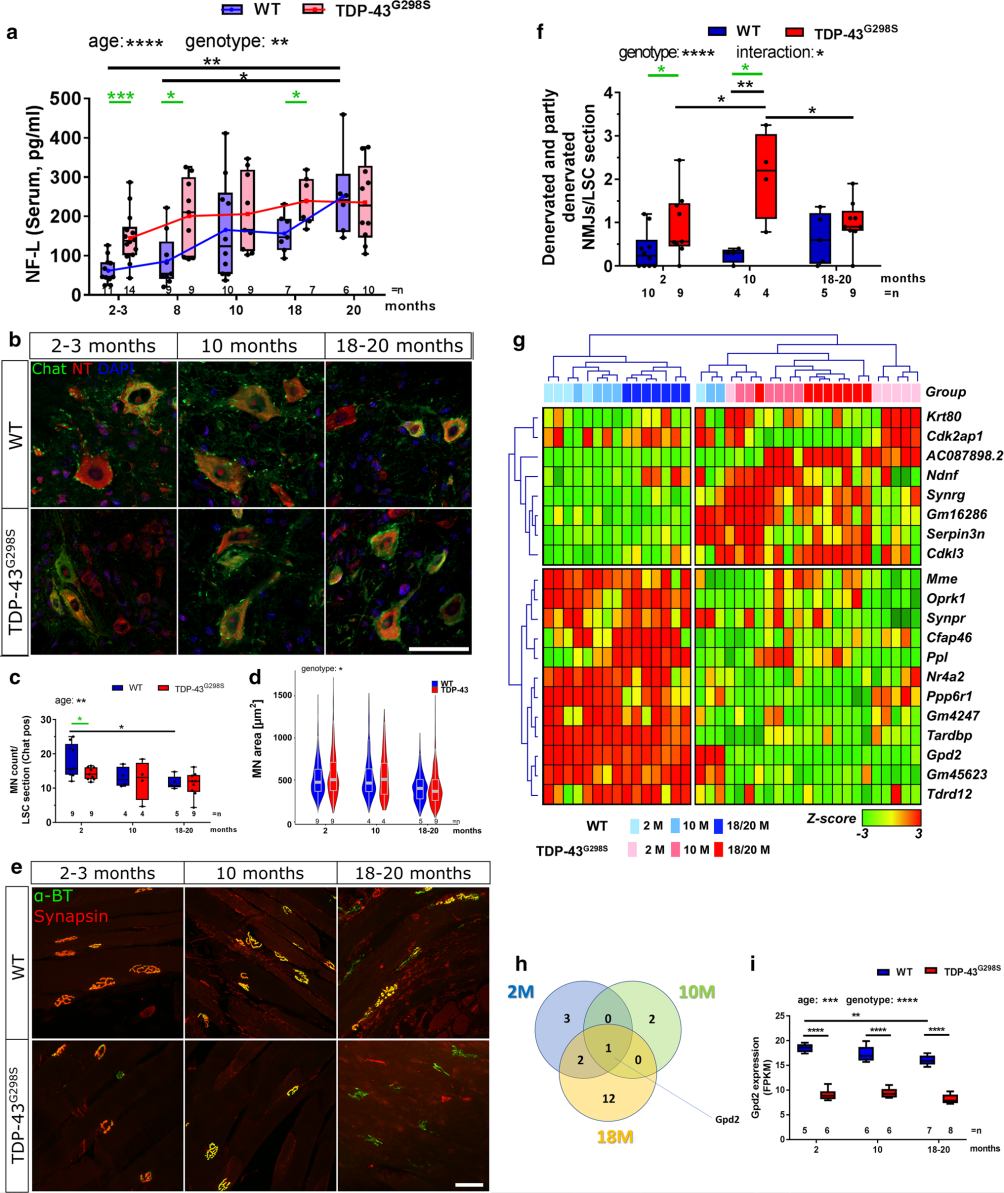
Age and disease dependent changes in TDP-43^G298S^ mice according to serum NF-L levels, NMJ integrity, MN count and area. **a** Serum NF-L levels were determined in WT and TDP-43^G298S^ transgenic mice at 2–3 months, 8 months, 10 months, 18 months and 20 months of age by single molecule array (Simoa) technology. Data distribution is shown as box plots (box showing the 25% and 75% percentile, with the line as median and the + indicating the mean, which is connected by lines, whiskers show min and max) *p < 0.05, **p < 0.01, ****p < 0.0001, Two-Way ANOVA with Tukey’s multiple comparison test. Significant differences between genotypes of one age group were determined by Mann–Whitney test (green) *p < 0.05, ***p < 0.001, *n* = 7–14, every time point reflects an end point measurement. **b** The number of motor neurons (MN) was determined in WT and TDP-43^G298S^ mice in the ventral horn of the lumbar spinal cord (LSC). Representative pictures of the counted areas of the ventral horn are shown. Neurotrace (NT, red) and choline acetyltransferase (Chat, green) positive cells were counted in 4–10 sections per animal (WT/TDP-43^G298S^: (2mo) *n* = 9/9; (10mo) *n* = 4/4; (18-20mo) *n* = 5/9). Scale bar represents 50 µm. **c** Statistical analysis of MN counts was performed by Two-Way ANOVA with Tukey’s multiple comparison test (*p < 0.05, **p < 0.01, ***p < 0.001, ****p < 0.0001). Significant differences between genotypes of one age group were determined by Mann–Whitney test (green) (*p < 0.05). Data are shown as box plots *n* = 4–9. **d** MN area was marked and measured in LSC sections of the mice using image J software. Using Two-Way ANOVA for statistical analysis age (***p < 0.001), genotype (*p < 0.05) and interaction of both (***p < 0.001) were significant. **e** The overlap of synapsin (red) and α-bungarotoxin (green) positive neuromuscular junctions (NMJs) were counted in the musculus quadriceps of WT and TDP-43^G298S^ mice. 9–14 pictures were analyzed per animal and representative images are shown. Scale bar represents 50 µm. **f** The amount of denervated (partly or complete) NMJs was determined. Statistical analysis was performed by two-way ANOVA with Tukey’s multiple comparison test. Significant differences between genotypes of one age group were determined by Mann–Whitney test (green), *n* = 4–10. **g** Transcriptomic profiling confirms pathologic changes in TDP-43^G298S^ mice already at 2 months of age. Gene expression was profiled by RNA-seq of total RNA isolated from the cortex of mice at all three age groups. Differential expression analysis identifies 20 genes which are differentially expressed in TDP-43^G298S^ mice at FDR *q* < 0.05 and robustly separate them from wild-type mice in a hierarchical cluster analysis (average linkage with Euclidean distance). **h** Analysis with relaxed FDR threshold (*p < *0.05, FDR *q* < 0.05) reveals the highest number of dysregulated genes in the 18–20 months group, but also dysregulated genes already at 2 months of age. **i** Gpd2 is significantly decreased in TDP-43^G298S^ mice at all ages and decreases with age in wild-type mice (Two-way-ANOVA with post-hoc Sidak correction, *p < 0.05, **p < 0.01, ***p < 0.001, ****p < 0.0001)

To study whether destructive processes in MN pathophysiology might be the underlying cause of NF-L level increase, the number and area of MNs in the ventral horn of the lumbar spinal cord were determined. Again, as also seen for NF-L levels, already at the age of 2 months TDP-43^G298S^ mice differed significantly from WT mice showing reduced MN counts comparable to the MN count of WT mice at older ages like 10 or 18–20 months (*p < 0.05, Mann–Whitney test and **p < 0.01 for age Two-Way ANOVA with Tukey’s multiple comparison test) (Fig. 1b, c). This MN loss in WT mice with age has been shown before [7]. These results support the idea that NF-L levels correlate with degenerating or damaged MNs. Since it is known that larger MNs are more affected in ALS [5], we measured the area of all counted MNs and found a significant decrease in MN cell body area in TDP-43^G298S^ mice with age compared to controls (Two-Way ANOVA for statistical analysis age (***p < 0.001), genotype (*p < 0.05) and interaction of both (***p < 0.001)) (Fig. 1d). Cell body swelling of MN in 27 months old mice compared to 3–6 months old ones has been reported previously [7]. In contrast to this, we did not observe increased MN area in old mice (max. 20 months old). Since we saw larger neurons in TDP-43^G298S^ mice already at 2 months of age we speculate that this type of MNs is highly vulnerable in ALS building the basis for MN degeneration already at this early time point. MN loss results in muscle denervation and atrophy. Muscle weakness of the limbs is one early and progressing symptom of ALS and integrity of NMJ is a cellular correlate. Denervation of NMJs was determined according to the overlap of the presynaptic synapsin with the postsynaptic α-bungarotoxin immunofluorescent signal (Fig. 1e, f). The images in Fig. 1e are representative images but the quantification in Fig. 1f illustrates the pooled data from denervated and partly denervated NMJs**.** TDP-43^G298S^ mice at 2 months as well as 10 months showed increased denervation compared to WT mice (*p < 0.05 Mann–Whitney test, **p < 0.01 Two-Way ANOVA with Tukey’s multiple comparison test and *p < 0.05 Mann–Whitney test comparison of only two defined groups).

To further characterize ALS related alterations in TDP-43^G298S^ mice, we performed immunohistochemistry and oberserved that the overlap between TDP-43 and nuclear staining is less pronounced in TDP-43^G298S^ tg animals than in WT mice (Two-Way ANOVA for statistical analysis showed significant changes for the genotype with *p < 0.05) (supplemental Fig. 1a, b). In addition, we conducted RNA sequencing of the cortex of these mice for all age groups and identified genes that were significantly altered in each respective group, even at the early age of 2 months (Fig. 1g). Further analysis of the RNA sequencing data showed six genes, which show clearly a genotype specific regulation (suplemental Fig. 2a). The Venn diagram of the most significant hits again illustrates the separation of the groups by age and highlights one very significant candidate in common, namely the mitochondrial protein Glycerol-3-phosphate dehydrogenase (Gpd2) (Fig. 1h), which is also dysregulated in the ALS SOD1 mouse model [1] as well as the wobbler mouse [2], a model of motor neuron degeneration. Gpd2 reliably differentiates between WT and TDP-43^G298S^ animals throughout all groups of age (Fig. 1i) and should be investigated in further studies. In addition the sequencing data revealed that TDP-43^G298S^ expression in our mouse model does not alter mRNA expression levels of Nfel although it is known that these are binding partners (supplemental Fig. 2b).

Together, the present study demonstrates that ALS TDP-43^G298S^ mice recapitulate elevated serum NF-L levels as seen in ALS patients. Simultaneously to NF-L elevation MN loss and muscle denervation were detected suggesting that NF-L elevation might be due to MN degeneration. We conclude that this mouse model is suitable for biomarker research in ALS with regard to the specific mechanisms involved in the disease associated release of NF-L.

## Supplementary Information

Below is the link to the electronic supplementary material.

Supplementary file1 (PDF 331 KB)
