# Supplementary material for: Increased NF-L levels in the TDP-43G298S ALS mouse model resemble NF-L levels in ALS patients
Source: Acta Neuropathol. 2022 May 18;144(1):161–4. doi: 10.1007/s00401-022-02436-1 (PMC9217825; doi:10.1007/s00401-022-02436-1)
Supplement: Supplementary file 1 — Supplementary file1 (PDF 331 KB) [file 401_2022_2436_MOESM1_ESM.pdf]

## Increased NF-L levels in the TDP-43$^{G298S}$ ALS mouse model resemble NF-L levels in ALS patients

**Supplement**

Supplementary Figure 1

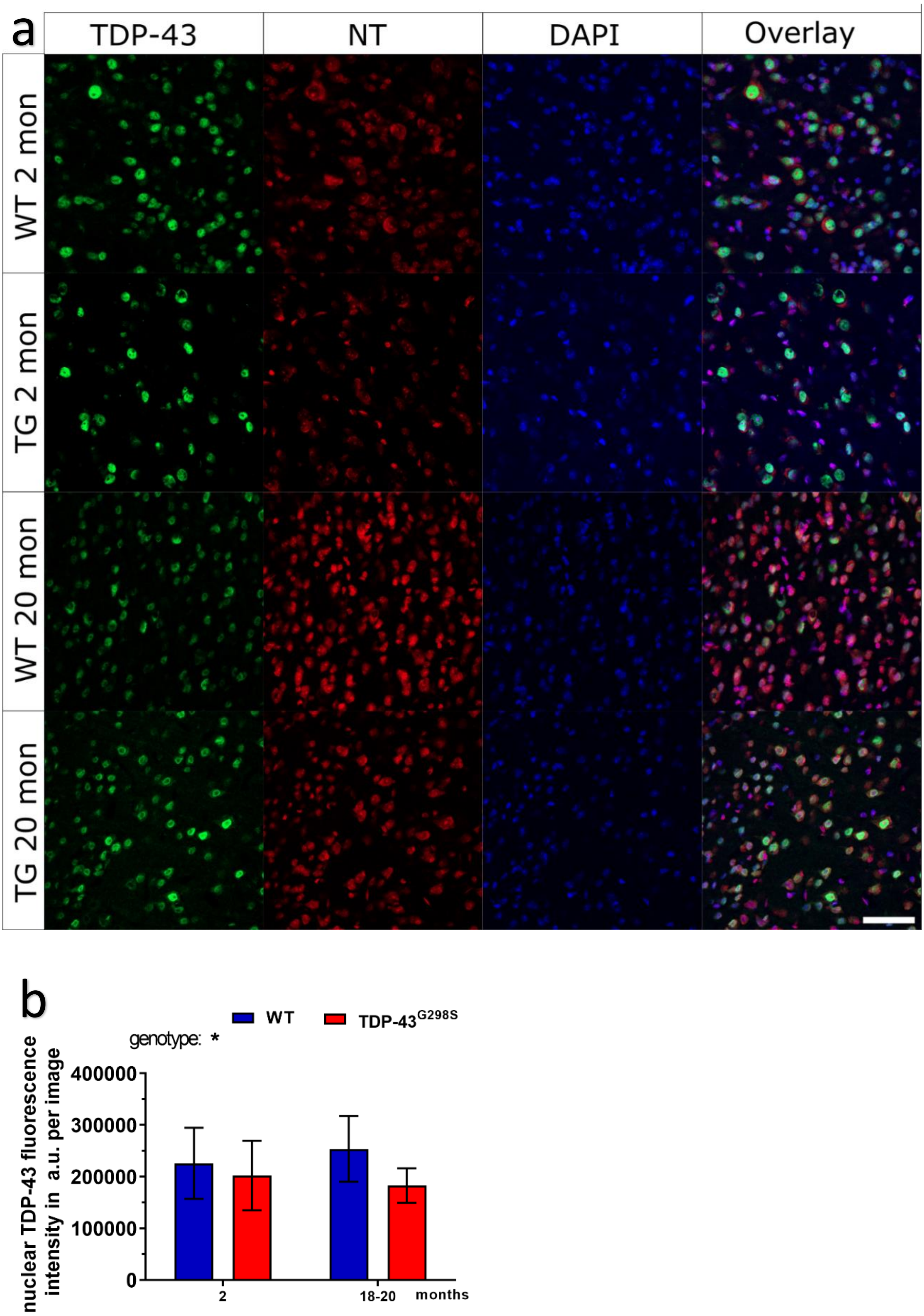

**Supplementary Figure 1 Transgene expression and localization in TDP-43$^{G298S}$ mice.** **a** Immunohistochemical stainings were performed in 2 and 20 months old WT and TDP-43$^{G298S}$ mice with 30µm thick coronal brain slices in a free floating approach. TDP-43 (green), Neurotrace (deep red) and DAPI (blue) were used to identify the TDP-43 protein distribution in cortical neurons. Representative images are shown, scale bar represents 50µm. **b** Quantification of nuclear TDP-43 fluorescence intensity is shown and statistical analysis was performed by Two-Way ANOVA (p*< 0.05).

# Supplementary Figure 2

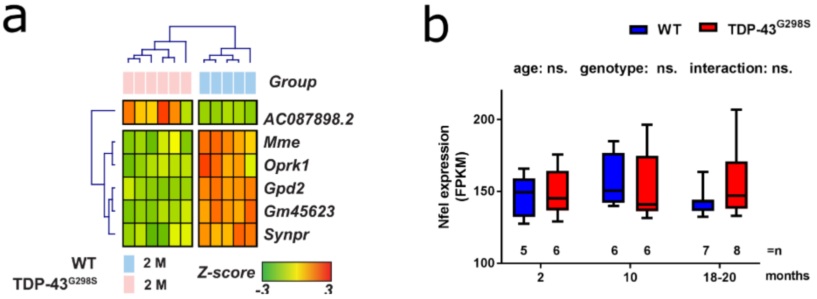


**Supplementary Figure 2 Transgene expression and localization in TDP-43$^{G298S}$ mice.** **a** Transcriptomic profiling identified genes, which robustly separate TDP-43$^{G298S}$ mice from WT mice already at two months of age. Gene expression was profiled by RNA-seq of total RNA isolated from the murine cortex. Differential expression analysis identifies 6 genes which are differentially expressed in the transgenic mice at FDR q<0.05 and robustly separate them from WT mice in a hierarchical cluster analysis (average linkage with Euclidean distance). **b** Nfel mRNA levels are unchanged in TDP-43$^{G298S}$ mice at all ages and compared to WT mice (Two-way-ANOVA with post-hoc Sidak correction, *p<0.05).
